# A Fast Shape-from-Focus-Based Surface Topography Measurement Method

**DOI:** 10.3390/s21082574

**Published:** 2021-04-07

**Authors:** Jona Gladines, Seppe Sels, Johan Blom, Steve Vanlanduit

**Affiliations:** 1 Faculty of Applied Engineering, University of Antwerp, 2020 Antwerp, Belgium; jona.gladines@uantwerpen.be (J.G.); seppe.sels@uantwerpen.be (S.S.); johan.blom@uantwerpen.be (J.B.); 2 Department of Mechanical Engineering, Pleinlaan 2, Vrije Universiteit Brussel, 1050 Brussel, Belgium

**Keywords:** 3D reconstruction, shape recovery, shape from focus, laser triangulation, optical dimensional metrology

## Abstract

Shape from focus is an accurate, but relatively time-consuming, 3D profilometry technique (compared to e.g., laser triangulation or fringe projection). This is the case because a large amount of data that needs to be captured and processed to obtain 3D measurements. In this paper, we propose a two-step shape-from-focus measurement approach that can improve the speed with 40%. By using a faster profilometry technique to create a coarse measurement of an unknown target, this coarse measurement can be used to limit the data capture to only the required frames. This method can significantly improve the measurement and processing speed. The method was tested on a 40 mm by 40 mm custom target and resulted in an overall 46% reduction of measurement time. The accuracy of the proposed method was compared against the conventional shape from focus method by comparing both methods with a more accurate reference.

## 1. Introduction

**Figure 1:**
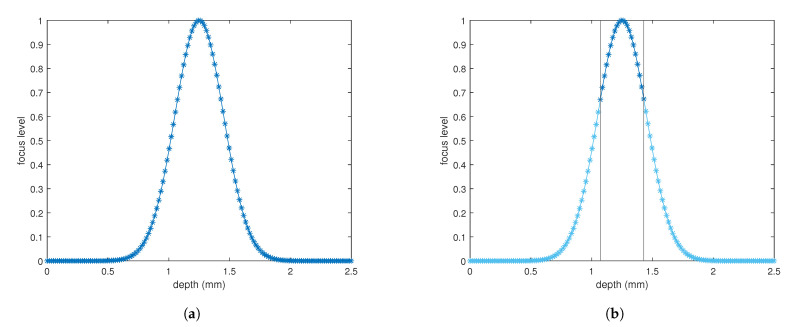
(**a**) Representation of a normalized pixel focus level Gaussian distribution when varying the focus point through the focal plain of a pixel. 0 equals fully out of focus, 1 equals maximum focus. Every point represents a captured image. (**b**) Thresholded profile, with sufficient points to determine pixel depth.

With the industrial integration of additive manufacturing into the standard production process comes the need for proper metrology on additive manufactured parts. Many techniques exist to recover the shape from an unknown object. Most techniques are developed for inspection and metrology of finished parts. To improve the quality of additive manufactured products it is of interest to develop these shape recovery techniques for use in at-line or even on-line metrology. In terms of precision and repeatability compared to other techniques like stereo vision or laser triangulation, shape from focus (SFF), otherwise known as depth from focus (DFF), is a good candidate for micrometer precision measurements [1,2,3]. Additionally, SFF has fewer problems with occultations compared to laser triangulation and has the possibility to create an all-in-focus image that could be used in the analysis for surface defects. Throughout the different techniques for optical shape recovery, SFF is somewhat of an outsider. Most 3D surface shape measurement techniques like Laser Triangulation [4,5,6], Stereo Vision [7], Photogrammetry [8] are based on triangulation. Laser triangulation is one of the most used techniques to date for inspection and metrology in industry [9,10]. Another popular technique is fringe projection, also known as structured light imaging, where projections of different sinusoidal waves on the target are combined to recover shape through phase unwrapping [11]. SFF tries to recover the camera–object distance based on the focus level of pixels [12,13,14]. To recover a surface profile with SFF, multiple images with very narrow depth of field are taken, while the focus plane is varied. This can be done using precision translation stages or with the use of electronically tunable lenses [2,15]. A calibration that determines the relationship between the optical focal point and camera distance is needed. Each of the resulting images is individually processed to measure the focus level for every pixel by means of a Focus Measure Operator (FMO). The focus level can be explained as a value for how much the specific pixel is in focus. The focus level of a pixel will normally follow a Gaussian profile similar to the schematic representation in Figure 1a. The maximum of this profile determines the point of maximum focus and by extension the depth of the specific pixel. Existing focus measure operators differ in processing speed, accuracy, robustness to noise and are extensively compared by Pertuz et al. [16]. Other approaches use Neural networking to recover the depth map [17]. Due to the high number of images (usually tens of images) and the large amount of processing required to create a depth map using SFF, the technique can be considered as time-consuming. The total measurement speed can be improved in multiple ways. Many of the existing focus measure operators are ideal candidates for optimization through processing on GPU. Algorithms based on image filtering through convolutions could be greatly sped up through parallel processing on GPU [18,19]. A second possibility to improve measurement speed would be to reduce the number of required images to produce a depth map. By limiting the image capturing to a smaller range around the target depth, the total amount of images to capture and by extension the measurement time can be reduced (Figure 1b). In this paper, we propose a two-step approach of first using a fast 3D profiling technique like laser triangulation (LT) to provide coarse depth information to limit the number of images to capture with SFF. The solution we propose can improve the measurement time independent of the hardware used.

The following sections will first present the traditional method of shape from focus, i.e., the capturing of images and the processing of these images to generate a depth map. Next, the proposed method, combining coarse topography methods with SFF to speedup the SFF measurement process, will be discussed. Then last, the measurement results will be presented and discussed in the results and conclusion.

## 2. Methodology

For use as an on-line metrology technique, it is crucial that the measurement process is sufficiently fast, depending on the speed of the manufacturing process, in order not to slow down the manufacturing. As explained in Section 1, the state of the art in SFF measurements is time-consuming, caused by slow-moving translation stages for the focus variation and slow image capturing and processing due to the large amounts of data required.

### 2.1. Traditional Shape from Focus

**Figure 2:**
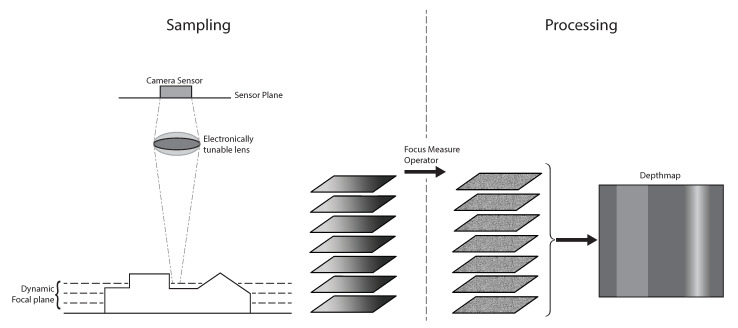
SFF sampling and processing using an ETL. The ETL is controlled to change the focal distance between every captured frame. All frames are then processed using the FMO, the result of this process is then converted in a depth map by either converting the image number of the image with the highest response to the FMO to a depth or by gaussian interpolation.

Section 1 describes how shape from focus measurements can be done using a precision translation stage, but also using Electronically Tunable Lenses or ETL’s. The sampling method for SFF with ETL’s is further explained by Figure 2.

ETL’s have an advantage over translation stages in terms of speed. The measurements in this paper were made with the Optotune EL-10-30C [15]. With at least 10 ms per image [2] they can be used at 100 fps imaging frequency to create a single depth map. A mechanical translation stage has to provide the upward or downward motion while keeping the camera or object stable in the lateral direction drastically reducing the translation speed. Since the ETL is only changing shape, the speed at which the focus can be varied is limited to the speed at which it can change shape. Our sampling rate was limited to an average of 50 fps due to a limitation in the camera driver. Combining the ETL with a 2× Finite conjugate microscope objective limits the tuning range in the depth direction to 2.5 mm. The ETL we have used is current-driven, meaning that an electrical current is applied to the lens to control its shape. To drive the lens we used the Optotune Lens Driver 4i, that can drive the lens in a range from 0 to 290 mA in 0.07 mA steps. This results in a maximum number of 4143 possible focus steps with a theoretical step height of 600 nm. Using this amount of steps for SFF imaging would result in extremely large datasets and therefore also slow measurement and processing speed. Large datasets do not lead to much-improved depth maps. We have compared the resulting depth maps generated from sampling in 50, 100, 150, 200, 1000 and 4000 steps of the same target section. from 200 steps upwards no significant change in measurement accuracy was seen. We opted to divide the tuning range of 2.5 mm in 150 steps of about 17 μm as a good compromise between measurement resolution and speed. The combination of camera, the Mako U-130b and the aforementioned optics resulted in a Field of View (FOV) of 4.27 mm by 5.35 mm. Combining a 1.16× magnification ratio with a 4.8 μm Pixel size from the camera, results in a lateral resolution of about 4 μm per pixel.

**Figure 3:**
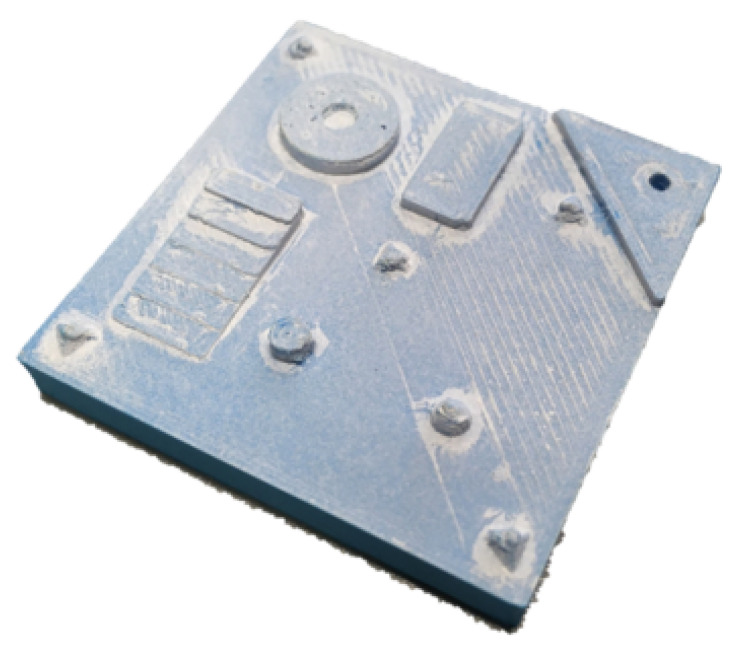
3D printed Imaging target of 40 mm by 40 mm with rectangular, spherical and cilindrical features.

**Figure 4:**
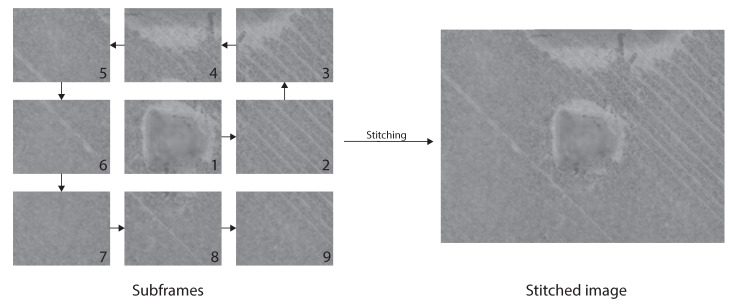
Principle of image stitching as implemented for this paper. Subframes (**left**) are taken counter clockwise with an overlap of 150 pixels and than stitched together to create an image of a larger area (**right**).

The relatively small FOV is thus required for μm accuracy measurements. For the purpose of this paper, we used a target with a size of 40 mm by 40 mm (Figure 3). In order to create depth maps of larger areas the target needed to be translated under the SFF camera system. We, therefore, used a pair of Zaber X-LSQ300B translation stages mounted orthogonally for XY-translation of the target. So to obtain a depth image of the full target multiple depth images had to be stitched together with some overlap for blending (Figure 4). Image stitching was performed based on the absolute translations made by the translation stages and not by feature detection and transformation.

**Figure 5:**
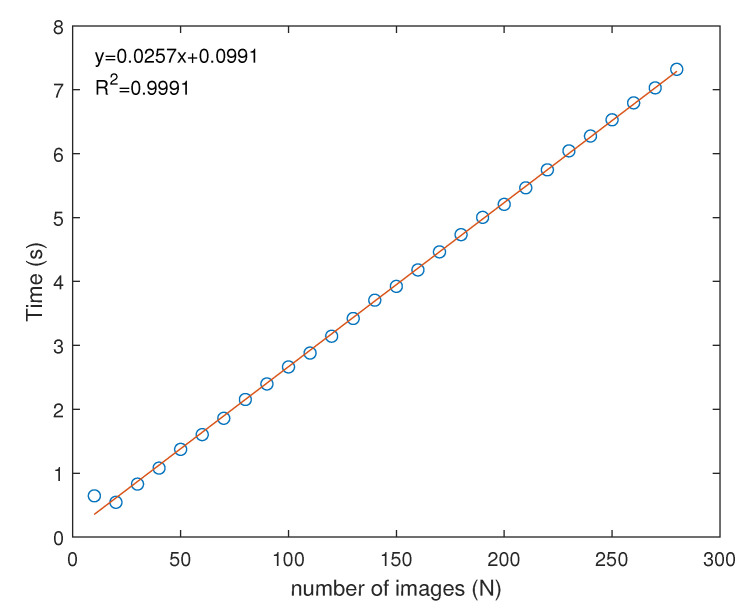
Processing time for a shape from focus data set (1280 × 1024 pixels) reduction to depth-map using the Modified Gray Level Variance (GLVM) focus measure operator in function of the number of images (N).

To create the depth-maps from the original images, we have used the Modified Gray Level Variance (GLVM) implementation in Matlab by Pertuz [20] and tested the processing speed for a different amount of images. The results of this can be seen in Figure 5. From this graph, it is clear that the processing time has a linear relationship with respect to the number of images.

Since the pixels sharpness value follows a Gaussian profile (Figure 1), it is possible to fit a Gaussian function during processing for every pixel and thus improve the depth resolution of the images [2]. This processing method was not used for this paper as it slowed down the processing by a considerable amount. Optimization of this process is another candidate for measurement time improvements. Therefore our depth resolution is limited to the 17 μm steps as described above.

### 2.2. Two-Step Shape from Focus

As described above, the conventional method for shape from focus has a lot of benefits for use in additive manufacturing but is slower than other topography methods because of the large amount of data capturing and processing needed for it. By comparison, for stereovision, a minimum of two images is required to produce a disparity map albeit with more complex processing. For laser triangulation, all processing complexity can be accounted for by calibration which results in a direct conversion from the reflected laser line to the actual height of an object. By limiting the amount of data to capture and process, SFF’s measurement speed can be improved invariant of the hardware used. This can be done by limiting the data capture to the depth range of the object’s section that is in the field of view of the system. So, instead of capturing images across the whole 2.5 mm range, data capture is limited to a smaller range, e.g., 1 mm. For unknown objects, it is impossible to predetermine this smaller range with the SFF system alone. Another topography method capable of creating depth-maps much faster but also coarser can be used to get a rough estimate of the object’s profile. The lower accuracy of the coarse depth map can then be translated to a margin around the estimated depth range to reduce the possibility of incorrect measurements.

**Figure 6:**
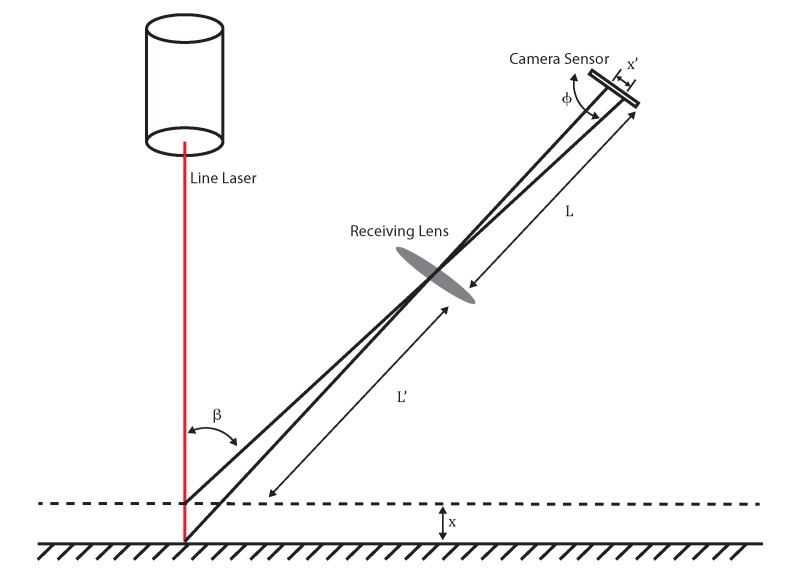
The principle of laser triangulation, where the position of a reflected laser line on a camera sensor is a function of the profile height. Based on an image from Sun B. and Li B. [22].

A possible topography method to use for the purpose of creating rough depth-maps is Laser triangulation (LT) also known as Sheet of Light (SoL) or laser ranging sensors [21], but any other method for fast creating a coarse profile can be used. Our setup was built similar to Figure 6 around a PhotonPhocus MV1-D2048-3D04 3D camera with a 12 mm lens, a line laser and a zaber X-LSQ300B translation stage for the third dimension. The camera was placed at a 15° angle to the laser line.

*L* and $L^{\prime }$ are the lens to object and to sensor distance, ϕ is the angle between the lens and the camera sensor which is 90° for our setup, β is the angle between the camera and the laser line. *x* and $x^{\prime }$ are the physical change in object height and its corresponding change in the laser spot on the sensor. The trigonometric relationship between *x* and $x^{\prime }$ is determined by [22]

(1) $$x=\frac{Lx^{\prime }sin\left(\phi \right)}{L^{\prime }sin\left(\beta \right)-x^{\prime }sin\left(\phi +\beta \right)}.$$

If we take $x^{\prime }$ equal to the camera sensor’s pixel size of 5.5 μm we can calculate the theoretical depth resolution of our setup. For us, this results in a theoretical limiting depth resolution of 20 μm for the LT setup. The real depth resolution is much worse as it is also influenced by laser scattering, optical aberrations and precision of calibration. The actual depth resolution was about 50 μm. The lateral resolution of the LT setup is defined by the camera resolution, the FOV of 50° and the step size of the translation stage. For our setup, we estimate the lateral resolution at 100 μm. A drawback of using laser triangulation to provide a coarse depth map are occultations. Because the camera is at an angle to the laser beam, occultations of the laser reflections on the target occur. The greater the angle, the bigger these occultations. So, height resolution of the LT system is always a trade-off with the occultations that will cause some areas to have no data. This can be resolved by placing multiple cameras. This is beyond the scope of this paper, so it was not included in this research. With the LT setup it was possible to scan the complete 40 mm-by-40 mm target at a low resolution in 45 s. With a minimal amount of processing, the point cloud generated by the LT setup can be converted to a coarse depth map.

**Figure 7:**
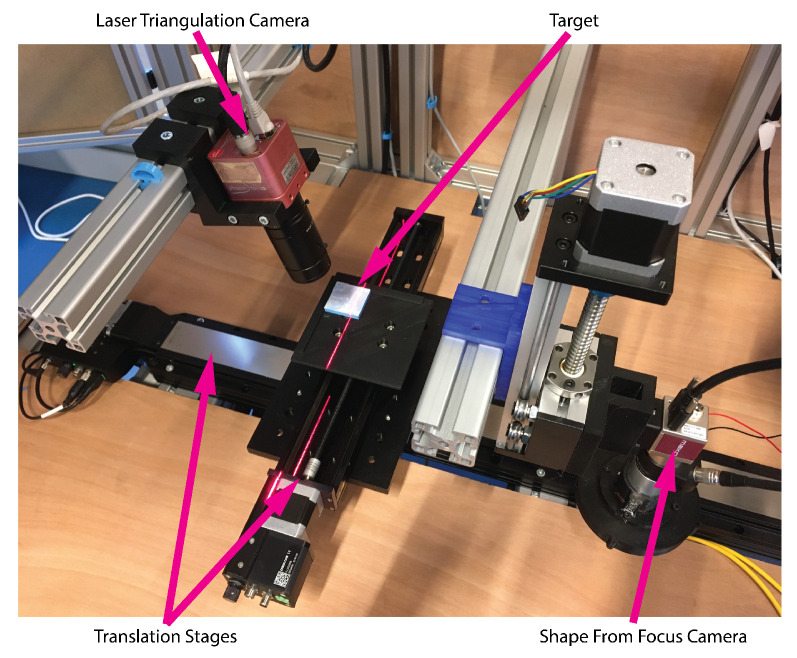
Image of the measurement setup with two 3D measurement systems and with the target on a sample plate mounted on top of two translation stages for xy-movement of the target.

The complete measurement system is thus composed of two individual measurement systems. One LT system for coarse measurements and one SFF system for precision measurements. The translation stages are common to both systems (Figure 7). As SFF requires optics with a small FOV and small depth of field (DOF) and Laser triangulation requiring optics with large FOV and big DOF it is not possible to combine the measurement systems using one camera and shared optics. Therefore, it was required to also calibrate both the SFF and LT system in terms of their extrinsic location.

**Figure 8:**
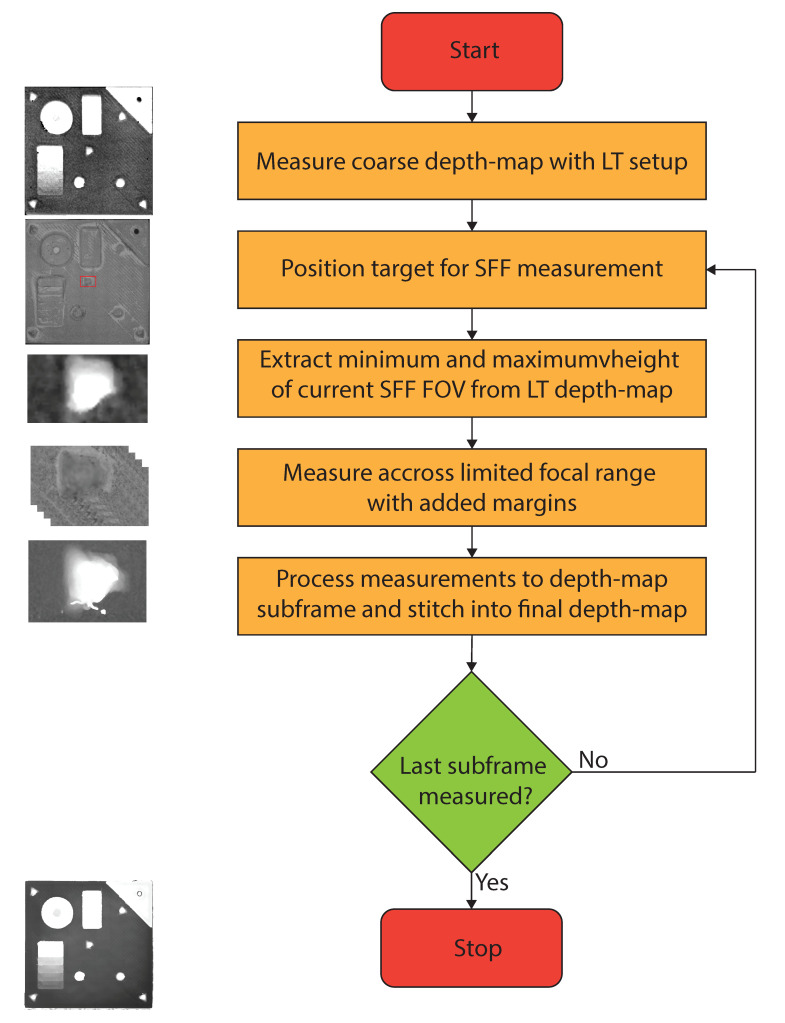
Flowchart of the measurement principle of combining laser triangulation (LT) and shape from focus (SFF) techniques to speed up stitched SFF measurements.

The measurement principle is very simple (Figure 8). Initially, the target is scanned by the LT system and a coarse point cloud is created. This point cloud is then converted into a depth map and scaled to the same resolution as the final SFF depth map. The LT depth map is then cropped and transformed to match the final FOV of the complete target. Now, the current small field of view of the SFF camera can be extracted from the coarse depth map. Next, the information of the extraction from the coarse depth-map is used to determine the minimum and maximum depth boundaries for the SFF scan. With the conventional SFF method, the creation of a subframe would take 150 images measured over a range of 2.5 mm. With this two-step approach, this measurement range is thus reduced to e.g., 1 mm. So, instead of 150 images, only 59 images need to be captured and processed.

Once the section is scanned using SFF with the limited range, the reduced data-set is processed with the exact same focus measure algorithm. Next, the translation stages move the target, so another section of it is visible for the SFF system and the process restarts from selecting the same section on the coarse depth map generated by the LT system.

This approach combines the benefits of the precision method with the speed of any other method. Additionally, developing the coarse method like LT to have a similar resolution as the SFF system would require an equally small FOV and therefore an increase in measurement time. The addition of extra components to a measurement setup also requires extra investment. However, the additional cost of the extra components is estimated to be small compared to the total measurement cost of measuring for prolonged periods of time. The cost of the laser triangulation system is in the order of magnitude of 10,000 euro. Whereas the measurement cost of running this equipment is estimated to be a minimum of 100 euro per hour. After already 200 h of measurement time, the extra investment cost would already be earned back. Adding a second measurement system for improvement of the original system, can in certain situations be a limitation. e.g., in mobile applications, adding the second measurement system introduces a lot of extra complexity. This method was specifically developed to be used for at-line or on-line metrology purposes, where the added complexity is not an issue.

#### Process Parameters

The gain in measurement time with this proposed two-step approach is dependent on two important parameters. Firstly due to the lower accuracy of the coarse measurement, a margin must be applied to the boundaries set to limit the capturing range. For example, if the coarse measurement has a depth resolution of 50 μm the margin should ideally be at least 50 μm above and 50 μm below. If we apply this to the example given above the actual measurement range is 1.1 mm instead of 1 mm. So with the margin applied the total amount of images to be captured is 66 for 1 mm. A larger margin leads to less gain in measurement time.

The second important parameter is the ratio between the measurement range of the SFF system and the number of subimages of the full target that need to be measured across the full range. If a relatively flat object is measured, the gain in measurement time will be large because most of the subframes of the depth map will require a limited set of images to be created. If a very rough object is measured that requires every subframe to be measured across the entire measurement range, then there is no gain in measurement time.

## 3. Results and Discussion

Table 1 shows the imaging, processing and total measurement time for the SFF system with and without prior depth knowledge. It is obvious that the addition of a coarse depth map to reduces the measurement range of the SFF system has a benefit on both capturing and processing. Where the normal SFF process would require capturing and processing of 25,350 images, applying this principle reduced this to 14,411. An excellent reduction of a little over 43%. Since the average number of images for a section of the depth map is 85 instead of 150, the processing time is also reduced from 844 to 436 s, which is a reduction of about 48%. The reduction in processing time should be similar to the reduction in the number of images since the processing time scales linearly with the number of images (Figure 5). With an average frame rate of 50 fps, the capturing of 25,350 frames results in 507 s of capturing. It is reduced by 43% to 288 s for the proposed measuring method. The total measurement time becomes 1350 s for conventional SFF and 724 s for the two-step approach, which equals a total reduction of 46% in measurement time on the SFF system. If we would include the measurement time and preprocessing of the laser triangulation measurement, 45 s need to be added to the two-step approach. Then, the total measurement time is reduced from 1350 to 769 s, a 43% reduction compared to the traditional method.

**Figure 9:**
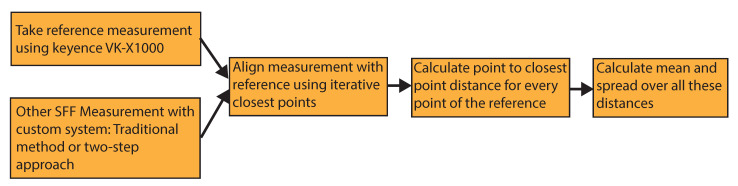
Comparison process of measurements of our own system with the reference measurements from the Keyence VK-X1000 with the CloudCompare software.

The reduction in measurement time would not be beneficial if it would also mean a reduction in measurement accuracy. To validate this, we performed reference measurements with a Keyence VK-X1000 using its shape from focus option. The reference measurements have an accuracy of approximately 500 nm and took about 3 h to complete due to the much higher resolution of the Keyence VK-X1000. The measurements of our system were then compared with these reference measurements using the software CloudCompare [23]. For the comparison, the depth maps were converted to point clouds. The respective point clouds were cropped to all show the exact same FOV of the target. After a coarse manual alignment of the point clouds, the alignment was optimized using Iterative Closest Points (ICP) [24]. After alignment, the point-to-point distance of a measurement to the reference was taken. This process is shown in Figure 9. The measurements were averaged over five individual measurements with a random offset applied to the starting position of the measurement to rule out any lucky measurements. For comparison, the same process was also applied to the point-cloud from the LT system.

**Figure 10:**
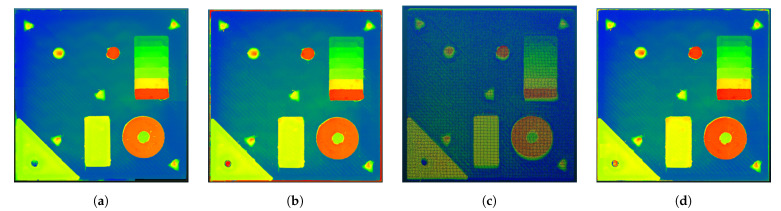
Point clouds generated from the different measurments (**a**) Keyence VK-X1000 reference, (**b**) shape from focus without thresholding, (**c**) laser triangulation, (**d**) shape from focus with thresholding from LT information.

Figure 10 shows the final depth-maps created by the different measurement systems converted to point clouds in CloudCompare.

**Figure 11:**
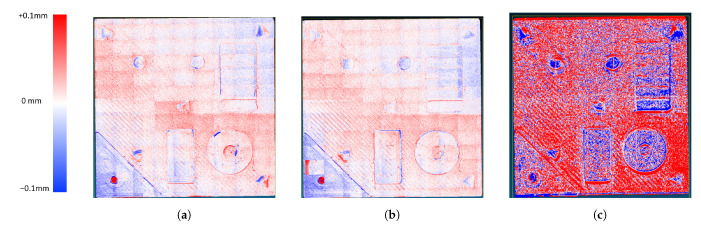
Comparison of the conventional SFF method (**a**), the proposed two-step approach (**b**) and the laser triangulation measurement (**c**) with the reference measurement.

From Figure 11 and the data in Table 2 it is clear that the application of the novel method had no negative influence on the accuracy of the SFF scan. The mean deviation from the reference of $0.3\times 10^{-3}$ and $0.1\times 10^{-3}$ mm has the same order of magnitude for both measurements. The same conclusion can be made for the standard deviation of the measurements, with 0.033 and 0.026 mm. The minor difference of 7 μm in standard deviation is negligible compared to the standard deviation of the laser triangulation measurement.

## 4. Conclusions

We have introduced a two-step approach to improve the measurement speed of shape from focus. We have shown that, by applying coarse depth information to shape from focus, the measurement time for stitched measurements can be reduced by limiting the measurement ranges of the individual sub-frames. This approach has led to an overall improvement in the measurement time of 43% on a test target. It has been shown that the proposed method does not impact the accuracy of the measurement. The method is independent of the hardware used. The total time reduction is, however, dependent on multiple parameters. First of all, the added margin to the limits set by the coarse measurement. The applied margin is limited by the accuracy of the fast 3D profilometry technique. Using a more accurate system for the coarse measurements allows us to use narrower margins and therefore a bigger reduction in measurement time. Secondly, the measurement time reduction is dependent on the ratio between the field of view of the shape from focus system and the height ranges of the target within this field of view. If the target has height variations over the full shape from focus range in every section to be imaged, the total reduction in measurement time can be zero. However, the field of view and measurement range of the shape from focus system could also be adapted or optimized for specific parts to provide the maximum reduction. Lastly, due to the lower resolution of the laser triangulation scan some objects of the target might go undetected. So those features will not be included while determining the measurement range for the shape from focus scan which might result in errors.

A possible improvement for this method would be to use the measurement from the LT system as an initial value on a point basis when improving the accuracy by Gaussian fitting on the measurement points. The LT measurement could also be used to determine the validity of the depth estimation by shape from focus. To further improve upon SFF as a technique it should be possible to speed up processing significantly with the use of GPU. Using higher speed cameras and a different way of controlling the ETL can possibly also lead to additional gains in measurement time.

## Figures and Tables

**Table 1:** Measurement and processing times for Conventional SFF and the proposed two-step SFF method. The processing method used was GLVM, implemented in Matlab R2019b [20] and run on a AMD Ryzen 3900× CPU.

Measurement	Total Number of Images	Imaging Time (s)	Processing Time on CPU (s)	Total Measurement Time (s)
Conventional Shape from focus method	25,350	507	844	1350
Two-step Shape from focus method	14,411	288	436	724

**Table 2:** Measurement result of comparison with reference, measured with keyence VHX-1000.

Measurement	Mean Deviation from Reference (mm)	Standard Deviation (mm)
Conventional Shape from focus	$0.3\times 10^{-3}$	0.033
Proposed Two-step approach	$0.1\times 10^{-3}$	0.026
Laser Triangulation Measurement	$2.0\times 10^{-3}$	0.120

## Data Availability

Not applicable.

## Abbreviations

The following abbreviations are used in this manuscript:

3D	Three Dimensional
DFF	Depth From Focus
DOF	Depth Of Field
ETL	Electronically Tunable Lens
FMO	Focus Measure Operator
FOV	Field Of View
GLVM	Modified Gray Level Variance
GPU	Graphical Processing Unit
LT	Laser Triangulation
ICP	Iterative Closest Points
MDPI	Multidisciplinary Digital Publishing Institute
ICP	Iterative Closest Points
PTC	Portable Calibration Target
SFF	Shape From Focus
STL	Stereo Lithography
